# Ethics and Effectiveness of US COVID-19 Vaccine Mandates and Vaccination Passports: A Review

**DOI:** 10.34172/jrhs.2022.81

**Published:** 2022-05-11

**Authors:** Alexa G. Canning, Kyleigh E. Watson, Katelyn E. McCreedy, John O. Olawepo

**Affiliations:** ^1^Department of Health Sciences, Bouvé College of Health Sciences, Northeastern University, Boston, MA, USA

**Keywords:** COVID-19 vaccines, Global health, Travel-related illness, Vaccine mandates, Vaccination passports

## Abstract

**Background:** The highest-income countries procured 50 times as many COVID-19 vaccines as low-income countries, a global health inequity that resulted in only 4.6% of the poorest 5th of the world receiving a COVID-19 vaccine. High-income countries are considering vaccine mandates and passports to contain the spread of COVID-19. This study is a curated discourse aimed at examining how vaccine mandates and passports may impact global vaccine equity from an ethics perspective.

**Study Design:** Narrative review adapted for a debate.

**Methods:** In November 2021, we conducted a review of studies examining global vaccine mandates for an upper-level global health course at Northeastern University, Boston, United States (U.S.). In total, 19 upper-level students, one research assistant, and one instructor participated in the data collection, analysis, and discussion.

**Results:** The review showed vaccine mandates are ethical and effective if autonomy-centered alternatives like soft mandates are first exhausted. Unwarranted stringent public health measures degrade public trust. In the U.S. alone, COVID-19-related deaths hovered above 300000 before COVID-19 vaccination began in mid-December 2020. Since then, the number of COVID-19 deaths more than doubled, despite the wide availability of the vaccine. For many low- and middle-income countries (LMICs) vaccines are not available or easily accessible. Global collaboration to facilitate vaccine availability in LMICs should be a priority.

**Conclusions:** It is essential to get as many people as possible vaccinated to return to some normality. However, vaccine mandates and passports need to be used only sparingly, especially when other options have been exhausted.

## Background

 As of December 1, 2021, there were more than 260 million cases of severe acute respiratory syndrome coronavirus 2 (SARS-CoV-2, referred to as COVID-19 subsequently) and 5.2 million deaths worldwide.^1^ The United States (U.S.) alone had 48.6 million cases, the highest number globally.^1^ This global health crisis persisted through global vaccine rollouts, as new variants emerged and many countries, including the U.S., are now experiencing a “third wave” of cases. This is despite COVID-19 vaccines that are effective at preventing severe illness and death, while also reducing the spread of the virus.^1^ In a study conducted by the U.S. Department of Health and Human Services, COVID-19 vaccination was correlated with a reduction of 265 000 new infections, a total of 107 000 hospitalizations, and around 39 000 deaths in the U.S. alone.^2^ The continued wave of cases on the global stage may be explained by the increasingly widening disparity in vaccine rollout between high and low-income countries. Over 7.14 billion doses of the COVID-19 vaccine were administered, yet only 4.6% of doses were administered in the poorest fifth of the world.^1^ Many doses are going to waste in high-income countries whose citizens refuse to be vaccinated.^1^ The People’s Vaccine Alliance found that high-income countries received 50 times as many vaccine doses as low-income countries, despite only having twice the population.^3^ As of September 2021, the U.S. alone discarded over 15 million doses.^4^ These issues raise important questions about global health vaccine equity.

 This study reports on the findings from a debate conducted in the PHTH 4120 course (Global Perspectives on Discrimination and Health) at Northeastern University, Boston, U.S., in November 2021. The course’s objectives were to explore global health disparities, identify causes of health disparities in marginalized populations and critically examine theoretical models for understanding discrimination and its relationship to health disparities. In this class, students were encouraged to think critically, analyze public health research, and practically apply learning to current topics in global health. The COVID-19 pandemic raises current and pressing global health vaccine equity concerns.

## Methods

 The study design was a narrative review to inform an academic debate on global vaccine mandates for an upper-level global health course debate at Northeastern University, Boston, U.S. In total, 19 upper-level students, one graduate research assistant, and one instructor participated in the data collection, analysis, and discussion of this study. The database search included Google Scholar, Northeastern’s Online Library, and PubMed. Studies published between 2019 to 2021, that were peer-reviewed and discussed vaccine mandates, vaccination passports, global health, and/or global vaccine equity were included.

 The debate was structured with four minutes for opening remarks and arguments, 3 min for rebuttal, 2 min to answer questions, and an additional 2 min for summation. Each debate lasted about 30 min, questions inclusive. The review and debate analyzed vaccine mandates and vaccination passports related to the state of the COVID-19 pandemic as of November 2021.

## Results and Discussion

 For the debate and discussions, our team reviewed 50 journal articles and websites. These readings were then curated and organized as discussion points.

## Topic One: Should the Government of the United States Mandate Vaccinations for COVID-19?

 Though it is known that vaccination is one of the most effective ways to control the COVID-19 pandemic,^5^ vaccination rates remain low in many low-income countries.^6^ Many countries are deliberating on implementing vaccine mandates to increase vaccination rates and, ultimately, achieve public health preventative goals. In the U.S., proof of vaccination is mandatory in certain settings, such as workplaces, schools, restaurants, or certain events in states that did not block U.S. President Joe Biden’s vaccine mandate.^6,7^ The Supreme Court will hear oral arguments in January 2022 over the legality of President Biden’s vaccine mandate.

###  In Favor: The U.S. Government Should Mandate COVID-19 Vaccinations

 Those in favor of a vaccine mandate cite the overarching need to protect the health of all Americans, particularly children and immunocompromised individuals. Additional arguments in favor of the mandate discuss how prior vaccine mandates were successful and highlight the economic and social benefits of a mandate.

####  A Duty to Protect Key Populations

 Before the week of November 8, 2021, children younger than 12 in the U.S. were not yet cleared to receive COVID-19 vaccines.^8^ To achieve public health goals and protect those who are unable to protect themselves, eligible individuals should receive the COVID-19 vaccination.^9^ Without a mandate, these select populations would be at risk of serious harm due to their inability to receive the vaccine. Vaccination is often perceived as an individual choice; however, it transcends personal choice when the infection is highly transmissible and can result in severe disease, hospitalization, and death if contracted by these populations.

####  The State of Vaccine Mandates

 Vaccine mandates are not new: historically, mandates began in 1806, when Elisa Bonaparte of present-day Italy mandated the smallpox vaccine for newborn babies and adults.^10^ In U.S. history, the right to mandate vaccines was established after the 1904 Supreme Court case *Jacobson v. Massachusetts* ruled that the smallpox vaccine could be required for all adults in Cambridge, Massachusetts.^11^ Refusal of the vaccine in 1904 resulted in a five-dollar fine.^12^ Fines are still implemented today for noncompliance; President Joe Biden stated that fines of up to $13 600 per violation will be issued to non-compliant employers.^10^ Since 1904, many other vaccine mandates have been implemented in schools, for federal employees, and employees in healthcare settings. As a result of the Child Immunization Initiative, which aimed to immunize children against seven diseases, all 50 states have laws that require vaccination for entering public schools.^13^ Now that the COVID-19 vaccination is safe and effective, implementing a mandate would lead to the protection of a large portion of the population against COVID-19, thereby approaching herd immunity.

####  Economic and Social Benefits of a Mandate

 COVID-19 has negatively impacted the U.S. economy, as evidenced by rising unemployment rates, business closures, decreased industrial production, and decreased household spending.^14^ Between January 2020 and July 2020, the unemployment rate reached 10.1% from a previous 3.6%,^15^ and the National Restaurant Association reports that the restaurant industry lost more than $120 billion in sales, in the first three months of the pandemic.^16^ The U.S. economy is not the only economy to suffer; the Brookings Institute reported that over 90% of the global economy faced a decline in per capita Gross domestic product.^14^In short, COVID-19 initiated a global economic crisis. The encouragement of people to receive the vaccine can help stabilize the economy and avoid another lockdown period. While mandates do not directly force people to receive a vaccine, they do limit people from attending school, going to work, traveling, or attending certain events. The Centers for Disease Control and Prevention (CDC) states that for those who receive both doses of the vaccine, normal pre-pandemic activities could resume.^17^

###  In Opposition: The U.S. Government Should Not Mandate COVID-19 Vaccinations

 This review found that the most well-studied and compelling arguments against a vaccine mandate revolve around its potential effect on public trust, personal autonomy, and managing special considerations for populations, such as children and healthcare workers.

####  Public Trust

 The potential effect of a vaccine mandate on public trust in policymakers and the scientific community should be fully explored. If a mandate were to decrease public confidence and trust for some populations, adherence to future potentially more life-threatening public health measures may be hindered. Widdus and Larson, in a published commentary on why vaccination programs fail, mention that these programs do not wholly understand the perceptions, values, and beliefs that guide people’s decision on whether to get vaccinated or not.^18^ Some people hold religious, philosophical, or political beliefs that affect their vaccine acceptance, while others, particularly disadvantaged communities, may have vaccine hesitancy or suspicion of intentional harm based on historic mistreatment by public health researchers.^18^ We must indeed move closer to achieving herd immunity; however, vaccine mandates and messaging can have unintended consequences for future public health issues. As a case in point, in some countries, people remember being physically forced to be vaccinated against smallpox, a memory that influences the vaccine hesitancy of those individuals today.^18^ Before the COVID-19 outbreak, overall vaccination rates were not increasing significantly, likely due to distrust in government authorities, which has only risen in recent years.^18^ It is more difficult to build public trust than it is to lose it; therefore, governments must weigh this concern when considering the imposition of a mandate on their citizens.

####  Personal Autonomy

 Before vaccine mandates are implemented, authorities must consider other options to achieve vaccine uptake. If there are methods that are less intrusive and do not infringe on personal autonomy, those methods must be used first. If a mandate is put in place before these less coercive measures are taken, the mandate has less ethical justification.

 A “soft mandate” is one approach to improve vaccine uptake that is less intrusive to personal liberty, compared to a federal mandate. Colleges, such as the University of California San Diego (UCSD, San Diego, U.S.), required unvaccinated students to be tested weekly for COVID-19 when they first rolled out a mandate.^19^ The UCSD later moved to a formal vaccine mandate without the testing exemption. However, this scenario allowed personal bodily autonomy to be maintained for as long as possible, while also monitoring the safety of the university population. Employers also can create positive incentives for their workers. For example, Target (Minneapolis, U.S.), among other corporations, will pay workers an equivalent of four hours of work to get vaccinated. Lyft (San Francisco, U.S.) also created free rides for people to get to their vaccination appointments. Those who are vaccinated get to reap these benefits which do not restrict personal liberties.

 Public health education that is accessible and non-stigmatizing is another approach that should be prioritized before a mandate. A mandate can undermine bodily autonomy and will push people further from the government and scientific community. Measure to educate people about the vaccine can increase its uptake.^20^ Unvaccinated people often report not understanding the importance of vaccination or feeling that they are unsafe or ineffective.^20^ Educational campaigns are one of the evidence-based solutions recommended by the CDC to help combat these beliefs.

####  Select Populations

*Schools*. Early in November 2021, vaccination against COVID-19 for children aged 5 to 11 years was authorized.^8^ Still, imposing a mandate for this age group and children under five involves many considerations. While it is true that many schools require proof of vaccination against diseases, such as measles, mumps, rubella, and polio, to attend school, these vaccines have been in use for decades, with widespread use beginning in the 1960s and 1970s.^13^ The COVID-19 vaccine differs in that it is approved under emergency use, and evidence is changing especially concerning its effectiveness against COVID-19 variants. The mandate for children to receive the vaccine has the potential to undermine public trust and raise considerations about sufficient supply and access.

*Healthcare Settings*. Healthcare workers have more frequent exposure to COVID-19 than the general population, making them a population where a vaccine mandate would be seriously considered. The imposition of a vaccine mandate on healthcare workers can have, and already has had, negative implications on the workforce. Healthcare workers are still dealing with an overburdened health system due to the COVID-19 outbreak. The mandate of vaccines results in healthcare workers quitting their jobs which will further compound the shortage of health workforce^21^ and potentially impact patient safety. Many organizations, such as Indiana University Health (Indianapolis, U.S.) and the Henry Ford Health System (Detroit, U.S.) had employees who voluntarily resigned from their position instead of getting the vaccine.^22^

## Topic Two: Should Countries Develop Vaccination Passports?

 The COVID-19 pandemic raises questions on the benefits of vaccination passports for international travel. This would entail the presentation of a physical copy of a vaccination passport or a digital health pass on a smartphone before international travel. While some countries employ this domestically and internationally, there is debate on its usefulness and accessibility globally.

###  In Favor: Countries Should Require Vaccination Passports

 Vaccination passports provide a potential tool for preventing the cross-border transmission of COVID-19 during international travel. Countries who choose to initiate this policy will significantly reduce the risk of international travelers infecting their citizens with COVID-19. Additionally, by restricting the ability of unvaccinated citizens to engage in international travel, it could increase the rates of vaccination within countries.

####  Decreasing the Spread of COVID-19

 Vaccination passport is a useful approach to reduce the spread of COVID-19 internationally. A recent study reported that 10% of the COVID-19 cases in 102 of 136 countries were due to international travel when there were no travel restrictions.^23^With reduced 2020 travel volumes and international closures, still 74 of 136 countries saw the same percentage of imported cases.^23^ This data purports that countries can expect travelers infected with COVID-19 to arrive in the absence of travel restrictions. Passports could be one means of reducing the number of cases by only allowing people who are vaccinated to enter the country. Vaccination does not offer foolproof protection; however, data published by the CDC show that vaccine effectiveness in preventing the spread of COVID-19 ranges from 66.3% to 95%.^24^ Vaccination passports will drastically reduce the burden of cases and protect vulnerable citizens in countries that rely on tourism, while also allowing for vaccinated tourists to safely visit the country.

####  Increasing Vaccination Rates

 By requiring vaccination passports, countries may see an increase in their overall vaccination rates. Many countries are struggling to encourage citizens to get vaccinated. In France, only 41% of the population was fully vaccinated as of July 2021.^25^ This changed, however, when President Macron announced the requirement for vaccination passports to enter public places. The next day over 1.3 million French citizens signed up for the vaccine, and currently, the country has 68% of its population fully vaccinated.^26^

####  Israel: An Example of Effective Vaccination Passports

 A prime example of how vaccination passports can benefit a country is Israel’s Green Pass. The original mandate of the pass required individuals to be fully vaccinated to attend large gatherings or enter most public spaces. This was dramatically effective in reducing the number of cases in Israel and the country had over a 90% receipt of two-dose vaccination.^27^ This dropped the cumulative incidence of COVID-19 cases in Israel with vaccinated persons accounting for 11% of new COVID-19 cases, while the 10% of the population that was unvaccinated accounted for 89% of new COVID-19 cases.^27^ The country is now requiring a third booster shot to qualify for the Green Pass after it was discovered that there is a decreased immunity six months after a person’s second dose. While Israel did not require its citizens to be vaccinated, they did restrict those who chose not to be vaccinated from most public events. This method, if replicated by other nations, could act as one of the least restrictive means to maintain public health. Without the vaccination passport, which encourages citizens to be vaccinated before traveling or attending events, countries could see another wave of COVID-19, future lockdowns, and more deaths as travel begins to open back up.

###  In Opposition: There Is No Benefit to Requiring Vaccination Passports

 As of November 2021, only 40% of the world’s population is fully vaccinated and only 1.1% had received a booster dose.^28^ It is not proper to require proof of vaccination to travel when this would heavily restrict most of the world’s population. Rather than requiring passports, which discriminate against low-income countries that cannot afford the vaccine, countries should keep current precautionary measures in place to protect their citizens from the global spread of COVID-19.

####  Travel Disparities between High and Low-Income Nations

 High-income countries are much more likely to implement vaccination passports, compared to their low-income counterparts. Sixty-eight percent (68%) of high-income countries use vaccination passports, while this percentage drops to only 6.9% for low-income countries.^29^ The only two low-income countries implementing vaccination passports are Tajikistan and Uganda.^29^ The reason behind this large difference is most likely due to large disparities in vaccine access. High-income countries procured vaccines early even before their initial rollout, despite only making up 14% of the global population.^30^ The U.S. and European nations have more than five times the number of doses per 100 000 people, compared to low-income nations.^31^ Low- and middle-income countries (LMICs) are left with a disproportionately smaller supply of the world’s COVID-19 vaccination. While global coordination can help to address the spread of COVID-19, this global health disparity will only be amplified if countries plan to enforce vaccination passports. Prices for the vaccines vary; however, LMICs are burdened with higher prices per dose. Pharmaceutical companies are charging governments over 40 billion dollars above the production cost and are charging countries different amounts based on undisclosed negotiations.^32^ Uganda, for example, paid $8.50 per dose for the AstraZeneca vaccine, whereas, the U.S.’s cost was only $4.00 per dose.^33^ This is one of the potential reasons Africa has the lowest vaccination rate with only 2% of the population having received one dose, compared to 40% of the European population.^31^ With such disparities in vaccination rate, requiring a vaccination passport to travel internationally will further the inequities between citizens of high-income countries and LMICs.

####  Implementation Issues

 For countries to feasibly implement vaccination passports, they must prioritize global vaccination. This is a difficult task to undertake and there are already many implementation issues regarding vaccine distribution. Distribution and trade are country-specific, and diverse healthcare systems would have to be dealt with individually. Many LMICs lack infrastructure and resources, including limited healthcare workers, storage capabilities, transportation, and refrigeration.^34^ Already, millions of doses of COVID-19 vaccines expired due to handling and resource distribution errors.^35^ The administration of the donated vaccines requires critical steps, such as distributing information through public channels, promoting vaccine education, and developing public trust, all of which are poorly executed in many countries.^36^ Vaccines may be donated, but it does not mean vaccination will occur. Nantahala Nyabola, a political analyst in Nairobi, Kenya, told The Telegraph, “We are setting up African countries to fail with leftover vaccines,”^36^ when discussing donated vaccines. With the limited capacity for vaccine distribution to countries that need it most, it is illogical to expect these countries to implement vaccination passports for international travel.

####  Reduction of Safety Measures

 The implementation of vaccination passports may also create a false sense of security which leads to the relaxation of other safety measures to prevent the spread of COVID-19. Although vaccines decrease the spread of disease, no vaccine is 100% effective. Those who are vaccinated can still contract the virus and spread the disease even if it is at a lower rate. Researchers from Johns Hopkins University (Baltimore, Maryland, U.S.) report that 1 in 5000 vaccinated individuals experience a breakthrough case of the original virus with many more contracting illnesses from the Delta and Omicron variants.^37^ This number is likely an undercount since symptoms are less severe, and thus, individuals are less inclined to be tested when sick.^38^ Breakthrough cases present a considerable challenge to countries that require vaccination passports instead of testing measures because they are unable to protect against the virus entering their countries. According to the World Health Organization (WHO), it is expected to take years to reach global herd immunity through vaccination, and vaccination passports should not replace other safety measures, such as testing and wearing masks during international travel.^39,40^

###  Rebuttal 

####  Proponents of Vaccination Passports 

 While concerns made about vaccination passports are valid, implementation would be beneficial to those affected by the pandemic. There is no dispute over disparities in vaccination between high and low-income countries. While it is an unfortunate reality that countries must protect their citizens before the rest of the world, the faster that higher-income countries vaccinate their citizens, the faster that other countries can be assisted, and more vaccines will be available at lower costs. The COVID-19 Vaccines Global Access (COVAX) and the World Bank are currently arranging a subsidized cost-sharing plan with the Vaccine Alliance to allow LMICs to purchase doses at a highly discounted rate.^41^ This will provide needed access to vaccines, and the implementation of vaccination passports for international travel will be more equitable. Vaccination passports should be coupled with testing, physical distancing, and the use of face masks to keep society as safe as possible.

####  Opponents of Vaccination Passports 

 The arguments made by the proponents of vaccination passports were cogent; however, there are other factors to consider. Firstly, the argument that the installation of passports will increase rates of vaccination is not internationally uniform. A United Kingdom study published in September 2021 found that those who express lower baseline intent for vaccination are more likely to express a lower inclination to get the vaccine if vaccination passports were introduced.^42^ This hesitancy is most prevalent in specific regional and ethnic groups whose vaccination rates are exceptionally low; therefore, public health interventions should be cautious of introducing measures that may push these groups farther from accepting the vaccine.

 While vaccination is an important step to decrease the spread of the virus, it is premature to expect vaccination passports will be the most effective measure. With such a low proportion of the global population currently vaccinated, passports will only provide a false sense of security to most of the world. The U.S., whose access to the vaccine is one of the highest in the world, has not seen a decrease in cases and deaths since the vaccine implementation.^43^ This is due to an increase in activity without an increase in safety measures, such as isolation, masks, and testing. With the implementation of a passport, countries will only be exacerbating that false sense of security without a commensurate decrease in the number of cases.

## Conclusion

 The increase of globalization and connectedness, paired with the implications of climate change, make pandemics more likely to occur than in previous decades.^44^ We will continue to face challenges with adherence to public health measures, as well as interventions; therefore, discourse about the positives and downfalls of interventions is crucial when developing responses to future outbreaks that threaten public health. Public health professionals undertake a considerable effort to educate the public about positive and negative behaviors that can impact their health. A significant part of the work by public health professionals is the continuous engagement and communication about practices that lead to negative health outcomes. The process of preparing for this review and debate helped students, the future public health professionals, to develop their ability to read complex and often conflicting literature, understand and synthesize them, communicate the important findings via a compelling discourse, and give a rebuttal to contrary opinions, especially those not clearly supported by evidence.

 The discussion about vaccines during this pandemic raises several additional controversies. First, the need for boosters and the changing definition of ‘fully vaccinated people’ are controversies that arise automatically from our attempts to combat this pandemic.^46,47^ Second, the fast-spreading omicron variant also raises several questions about ‘how many doses of vaccine we will need’ and the profiteering of pharmaceutical companies.^48^ Finally, the current immigration blockade placed on several African countries, even for those fully vaccinated, is a topic for reconsideration.^49,50^How practitioners of public health and global health deal with these issues will go a long way to determine how the public continues to perceive the ‘invisible science’ of public health. Additionally, it influences the discourse around increased funding for a field that at best is acknowledged only during pandemics and at the least is ignored and severely underfunded, especially in LMICs. The challenges that public health faces can only be addressed by having these robust discussions and debates both for educating the public and training the new crop of public health practitioners, such as those in this Global Perspectives Course.

 In summation, after 19 upper-level public health students reviewed comprehensive literature on vaccine mandates and passports to prepare for this debate, clear lessons on global health ethics emerged. The discourse revealed that as many people as possible should get the COVID-19 vaccine. Vaccines are safe and effective and will return society to relative normality. In the U.S., COVID-19-related deaths hovered above 300 000 before COVID-19 vaccination began in mid-December 2020.^4^ Since then, the number of COVID-19 deaths more than doubled, even with the wide availability of the vaccine.^45^ We know vaccination against COVID-19 is the most effective method of preventing related deaths, but why do deaths continue to soar? This narrative review and debate explored current vaccine mandates and passports as tools for preventing the spread of COVID-19. The development of variants occurs when the virus can spread and mutate, especially in unvaccinated populations. This underscores the vital nature of global health collaboration to continue COVID-19 vaccination efforts globally in ways that are equitable, effective, and ethical, most especially by improving vaccination rates in LMICs.

**Highlights**

- Vaccine mandates are ethical if autonomy-centered alternatives are first exhausted.
- Deaths from hospital-acquired COVID-19 make employer mandates ethical.
- High-income countries should improve LMIC vaccine equity before vaccination passports.
- Without global vaccine equity, COVID-19 outbreaks will continue globally.

## Acknowledgments

 The Global Perspectives Course Group (Fall 2021) at Northeastern University (Boston, U.S.) contributed research to this review: Abbas, Mahnoor; Alves, Emily R.; Berry, Mireille S.; Blaylock, Yasmine L.; Di Gangi, Catherine I.; Dobbins, Grace P.; Fitzpatrick, Mason S.; Gnanaravi, Gilda F.; Hassan, Zakariyya; Huynh, Dalena; Johnson, Sarah R.; Kam, Emily; McLean, Charlotte A.; Mei, Xin Xin; Sun, Edwin; Wilson, Devan C.; Yoon, Hailyn.

## Conflict of interest

 The researchers declare no conflicts of interest.

## Funding

 Nil.
